# Editorial: Coding for Spatial Orientation in Humans and Animals: Behavior, Circuits and Neurons

**DOI:** 10.3389/fncir.2020.619073

**Published:** 2020-11-25

**Authors:** Desdemona Fricker, Mathieu Beraneck, Michele Tagliabue, Kate J. Jeffery

**Affiliations:** ^1^Integrative Neuroscience & Cognition Center, CNRS UMR 8002, Université de Paris, Paris, France; ^2^Department of Experimental Psychology, Institute of Behavioural Neuroscience, University College London, London, United Kingdom

**Keywords:** anatomy, physiology, model, head direction, grid cell, self-motion, vestibular, visual

The goal of this Research Topic was to bring together articles on spatial orientation, from behavior and brain wide circuits to single cell anatomy and the function of neuronal networks. This research is aimed at understanding how the brain integrates multisensory spatial information, using diverse experimental approaches in humans and in rodents, as well as *in silico* computational tools.

Articles in this collection address a broad range of aspects of spatial orientation. Human studies are especially challenging when it comes to understanding the transformation of sensory information into motor commands. Blouin et al. analyzed cortical activation during movement planning and suggest that a cognitive representation for planning arm movements after body motion is necessary to store spatial information before triggering movement.

Maintaining spatial orientation requires monitoring self-motion cues and it depends on the integration of visual, proprioceptive, kinesthetic, and vestibular information. Stahn et al. investigated spatial updating during parabolic flight maneuvers, showing that updating performance critically depends on gravity.

Spatial disorientation is a major risk for pilots and a frequent cause of aircraft accidents. Tamura et al. examine electrodermal activity as a biomarker to evaluate spatial disorientation in pilot candidates, and they suggest that it may give a useful correlate for the severity of motion sickness.

Spatial navigation abilities change throughout life. To help to elucidate the cerebral bases of spatial navigation deficits as we get older, the study by Ramanoël et al. evaluated the effect of aging, showing age-related differences in the functional and structural connectivity of the spatial navigation brain network, and in particular between low-level visual areas and high-level spatial areas.

To investigate the physiology and pathology of spatial navigation, healthy human subjects and patients with neurological disorders can be examined either in real space or in virtual reality (VR). Schöberl et al. reviewed the advantages and limitations of real space navigation testing and different VR-based navigation paradigms, and discussed their potential future applications in clinical neurology.

The perception of optic flow is a fundamental part of the spatial toolkit. Solari et al. experimentally tested speed perception for conditions in which only portions of the visual field were visible, and captured the features of the human perceptual data in a biologically-inspired computational model.

This Research Topic puts special emphasis on the understanding the neural basis of spatial orientation across different species. The vestibular system is particularly important as a highly preserved sensory detector of orientation. Smith offers a comprehensive review of the growing evidence for the role of the otoliths in spatial memory, in both rodents and humans. He points out that loss of otolithic function impairs normal spatial memory and also impairs the normal function of head direction cells in the thalamus and place cells in the hippocampus.

The Review article by O'Mara and Aggleton addresses spatial and memory research that has long been focused on the hippocampus and entorhinal cortex, in rodents and in humans. The authors present evidence suggesting that spatial signals originally identified in the hippocampus are also observed in subcortical regions and they argue that subcortical circuitry should be better addressed in contemporary theories of spatial processing.

Dillingham and Vann summarize the mixed evidence regarding the contribution of the head direction system to behavior. Their Perspective discusses the observation that lesions of the mammillary nuclei, which lead to a disrupted head direction signal in downstream brain areas, do not lead to impaired performance on spatial tasks.

The rodent model system is particularly useful for detailed circuit analysis with modern tools such as virus-mediated trans-synaptic tracing. A tracing study in mice by Bohne et al. revealed a polysynaptic circuit from the deep cerebellar nuclei to the hippocampus via the thalamus. This result strengthens the notion of the cerebellum's involvement in spatial navigation.

Honda and Furuta employed sparse viral labeling techniques in rats to examine projections of presubicular neurons. Their highly detailed anatomical reconstructions offer novel insights into the anatomical organization of how head-direction information reaches the medial entorhinal cortex.

Decoding of neural population activity is a popular approach to assess the dynamics of spatial signals. Xu et al. provided an overview of different approaches, comparing machine learning and statistical model-based decoding methods, for head direction signals in thalamo-cortical brain areas.

Jayakumar et al. applied computational modeling to investigate grid cells in medial entorhinal cortex. Their model of grid cell generation was based on a combination of directionally tuned oscillators and Hebbian learning, and showed that grid cells encode invariant properties of an environment.

Laptev and Burgess presented a model of parallel place and grid cell attractor systems that provides a better account of classic place cell experiments than a model with only one attractor. Their study supports the hypothesis that grid cells are responsible for performing path integration while place cells are receiving information about environmental cues and external landmarks. Importantly the model makes predictions that can be tested in future experiments.

We are impressed with the broad range of contributions included in this Topic, and pleased that scientists from all walks of spatial orientation research chose to publish in this volume. We thank the authors for their contributions, and are grateful to the reviewers for their efforts.

## Author Contributions

All authors listed have made a substantial, direct and intellectual contribution to the work, and approved it for publication.

## Conflict of Interest

The authors declare that the research was conducted in the absence of any commercial or financial relationships that could be construed as a potential conflict of interest.

